# Introduction of a joint physiology-pharmacology capstone project in a premedical master’s program

**DOI:** 10.3389/fphys.2026.1871233

**Published:** 2026-07-01

**Authors:** Qing Zhong, Rachel M.A. Linger, Jacquelyn Waller, Joel A. Roberts

**Affiliations:** 1 Department of Biomedical Sciences, Rocky Vista University College of Osteopathic Medicine, Ivins, UT, United States; 2 Department of Biomedical Sciences, Rocky Vista University College of Osteopathic Medicine, Englewood, CO, United States; 3 Department of Biomedical Sciences, Rocky Vista University Montana College of Osteopathic Medicine, Billings, MT, United States

**Keywords:** capstone project, drugs, dual campuses, evaluation, feedback, joint physiology-pharmacology capstone project, master of science in biomedical science, reflection

## Abstract

**Introduction:**

Capstone projects are active and integrative, compared to passive learning from didactic lectures. Although some healthcare programs utilize these modalities, capstone projects have not been reported in a premedical master’s program. We introduced a joint physiology-pharmacology capstone project in a premedical master’s program.

**Methods:**

The joint capstone project was introduced in Spring 2022 across dual campuses with a total of 72 students. Students were divided into teams, and each team selected a disease-drug pair from a list of faculty-provided options. Students investigated the disease-related physiologic changes in multiple organ systems, general treatment strategy, novel drug development, and the new drug’s mechanism of action and side effects. Finally, each team generated a slide set and delivered a presentation. Each student was required to submit a reflection about the capstone project. The Block 4 Physiology exam assessed learning objectives derived from the core concepts presented in the students’ capstone projects. At the end of the semester, an anonymous survey collected students’ evaluations and feedback.

**Results:**

Student performance on the joint capstone project was excellent, with an average of above 93.5% on all physiology, pharmacology, and common domains. The mean performance on questions related to capstone diseases (self-learning) was similar to the average performance on the teacher-taught content in the Block 4 exam of the Physiology course. There were 72 (100%) responses to the evaluation survey. The majority of students (77.8% - 88.9%) agreed and strongly agreed that the joint capstone project facilitated self-directed learning of the pathophysiology, clinical trials, drug development, and therapeutic management of diseases. Students felt strongly that the joint capstone project enhanced the integration of physiology, pharmacology, and diseases. Teamwork was highly appreciated by students as well. In students’ reflections, integration, teamwork, and future application were the most common themes.

**Conclusion:**

A joint Physiology-Pharmacology capstone project in a premedical master’s program could be an effective and efficient strategy to enhance premedical students’ ability to integrate physiology, pharmacology, and disease. This project cultivates self-directed learning and teamwork.

## 1 Introduction

A ‘capstone’ originally referred to the final stone or brick that an architect would lay on a building. In educational contexts, a capstone project describes a culminating assignment that helps students learn how to find and analyze information and how to apply the information to solve a real-world problem (Wuller, 2010). These projects can be completed in various forms, including a multimedia presentation, film, performance, or paper. Capstone projects have become an integral part of some university degree curricula and have been used in healthcare education, such as for pharmacy students (Wuller, 2010; Patel et al., 2015), medical students (Chamberlain et al., 2020), students in master’s programs of health and biomedical informatics (Cox et al., 2021), students in specialized undergraduate biology programs (Goodman et al., 2015), and students in undergraduate physiology majors (Julien et al., 2012). But, so far, there is still no report of the capstone experience in pre-medical post-baccalaureate programs. Pre-medical post-baccalaureate programs are in a unique position to prepare students for success in entering medical schools, especially as some provide pathways into medical schools that bypass traditional medical admissions processes (AAMC, 2018). Graduates of such programs accounted for 14% of matriculants of medical schools in the United States in 2017 (AAMC, 2018).

Here, we introduce a capstone project in a Master of Science in Biomedical Science (MSBS) program, which is a premedical post-baccalaureate program whose graduates earn a master’s degree in Science. This 10-month master’s program at Rocky Vista University began on the Colorado campus in 2016, subsequently expanding to an additional campus in Utah in 2020. This master’s program intends to better prepare and recruit premedical students for medical or health-related study. Similar programs report improvement in medical students’ performance and surgical residency placement (Johnson et al., 2017).

The joint Physiology-Pharmacology capstone project was introduced in Spring 2022 across dual campuses.

In this study, we will present the effectiveness and lessons from this joint capstone project. Investigators hope that this project serves as a successful example of an interdisciplinary capstone project for other premedical master’s programs and medical schools.

## 2 Methods

### 2.1 Ethical approval

This project was approved by the Rocky Vista University (RVU) Institutional Review Board (IRB). The study was determined to be exempt by the IRB (IRB#2022-141).

### 2.2 Participants

All students in the MSBS Class of 2022 participated. There were 40 students on the Englewood, Colorado campus, and 32 students on the Ivins, Utah campus.

### 2.3 Joint capstone project design

The 10-month premedical MSBS program at Rocky Vista University was launched in 2016. Two 4-credit Physiology courses spanned two semesters (Fall and Spring), while a 3-credit Pharmacology course was delivered in the Spring semester.

In this joint capstone project, each student team is required to investigate a disease paired with a drug, generate a slide set (or PowerPoint), and deliver an oral presentation. Assessment is based on rubrics for physiology, pharmacology, and common domains. A total of 60 points is equally distributed to the Physiology course and Pharmacology course, with 30 points to each course. **Table 1** shows the requirements of the joint capstone project and credit distribution between physiology and pharmacology disciplines. The project spanned three months from the end of January to the end of April in 2022.

**Table 1:** The design of the joint capstone project.

Component	Physiology	Pharmacology
Total Points Assigned	30 points	30 points
Assignment Weight	10% of total course points (30/308)	14% of total course points (30/214)
Project Requirements	1. Draft PowerPoint slide set 2. Faculty feedback on draft 3. Final PowerPoint slide set 4. Oral presentation 5. Written reflection (each student, mandatory)	
After the oral presentation	Evaluation of Joint Capstone Project (each student, voluntary)	
Additional Assessment: Summative Assessment for each student	Pathophysiology aspects of Capstone-related diseases were tested in the Block 4 exam (final exam) (19Qs/total 30Qs) (6.2% of total course points (19/308))	None

PPT, PowerPoint; Qs, Questions.

The Capstone Project Guidelines stated, “For the Biomedical Pharmacology course, emphasis is placed on critical evaluation of the literature, teamwork, and synthesis of foundational pharmacology principles. For the Physiology II course, emphasis is placed on the case, pathophysiology, and review of key physiology concepts from Physiology I and Physiology II. At the conclusion of the project, students will complete a written reflection”.

For this joint project, in December 2021, the faculty generated a list of diseases linked with a novel drug that received FDA approval within the last five years in order to ensure every project included a relevant drug and a multi-system disease. An example of the disease-drug list is provided in Supplemental Information **Supplementary Table S1**.

Separate rubrics for the Physiology, Pharmacology, and Common domains were generated in December 2021. In brief, for the physiology domain, students are required to analyze pathophysiological changes in at least three organ systems of the selected disease and develop a clinical case for the disease. For the pharmacology domain, students are expected to discuss standard care for the disease and explain the selected drug’s development, pharmacodynamics, pharmacokinetics, and side effects. The common domain is to evaluate slide quality and citation accuracy, inclusion of health disparities related to the disease and drug, overall presentation performance, and students’ critical reflection on the project experience.

**Table 2** shows rubrics for the physiology domain and pharmacology domain, and **Table 3** shows the rubric for the common domain. Only the rating scale of 80-100% is shown. The full rubrics, including rating scales of 0-70% and 70-80%, are provided in the **Supplementary Information**.

**Table 2:** Rubrics for physiology and pharmacology domains with 80-100% scales.

Physiology domain (maximal 20 points)	Meets expectations (80-100%)	Pharmacology domain (maximal 20 points)	Meets expectations (80-100%)
Physiology Concepts (10 points max)	□Physiology information is thorough and concise □Includes pertinent concepts and explains them clearly and correctly □Includesat least three distinct physiology concepts from at least three different blocks	**Pharmacology Concepts** (10 points max)	□ Drug mechanism of action is thorough and concise □Includes pharmacokinetic parameters and explains them clearly and correctly □Adverse drug reactions are thoroughly and adequately discussed
Pathophysiology Integration (5 points max)	□Explanation of case findings and/or pathophysiology is correct and robustly connects the physiology concepts	**Review of Drug Development Literature** (3 points max)	□Background relevant to the history of drug development is adequately discussed □ Clinical trial (at least one Phase II or one Phase III) is adequately discussed
Case (5 points max)	□Case is plausible □Includes pertinent symptoms, vitals, physical exam, labs/studies, and treatment	**Standard of Care** (7 points max)	□Current standard of care is adequately discussed □ The chosen drug’s value in disease management compared to the current standard of care is adequately discussed

(Standards for 0-70% and 70-80% are available in **Supplementary Table S2** and **S3** in the Supplemental Information.).

**Table 3:** Common domain rubrics (80-100% scale).

Common domain (maximal 20 points)	Meets expectations (80-100%)
Written Communication and Visual Aids (5 points max)	□Visual aids contain appropriate amount of information, are of good quality, and effective at conveying content
Oral Communication (5 points max)	□The speakers are confident, articulate, and professional □Majority of information is memorized (not read from notes/slides) □Speakers respond adequately to questions from faculty & peers
Professional Behavior (2 points max)	□Speakers are professionally dressed □Respectful interactions with team, audience, and/or faculty □All assignments were completed by the due date
Learning Outcomes (2 points max)	□Presentation includes 3–5 specific, pertinent learning outcomes
Racial and Ethnic Health Disparities (2 points max)	□Racial and ethnic disparities relevant to disease and/or drug treatment are adequately discussed
Sources andCitations (2 points max)	□All non-original content is from a credible source and is appropriately cited using APA in text or endnote format □All images are appropriately cited (minimum of URL)
Timing (penalty points only)	□Presentation is over 22 min and less than 25 min
Individual reflection (2 points max)	□ Evidence of personal reflection that makes connections between learning and collaborative practice □ Rich in content; insightful analysis, synthesis, and evaluation

(Standards for 0-70% and 70-80% are available in **Supplementary Table S4** in the Supplemental Information).

Students were provided with a Capstone Project Guidelines document and an introductory lecture at the end of January 2022. The team members had been assigned in August 2021, with each team consisting of 5–7 students, and had worked together in the Physiology I course and other courses for one semester. Each team had two weeks to select a disease-drug pair. Teams had one month to submit the draft of slides, for which faculty members provided written feedback within 1 week. Students polished their slides based on faculty feedback and submitted the final slides in 3 weeks. The final team presentations were conducted in an auditorium over the next 10 days.

### 2.4 Assessment of student performance

#### 2.4.1 Team performance on the capstone projects

Three faculty members directed the joint capstone project: one Physiology (JR, Colorado campus) and two Pharmacology faculty members (QZ, Utah campus; RL, Colorado campus). JR, QZ, and RL provided feedback (indicating which components were missed) and graded the physiology, pharmacology, and common domains for slide drafts, the final slides, and the presentations, respectively. Except for students’ written critical reflections, which were assessed separately for individual students, all rubric domains were assessed at the team level.

#### 2.4.2 Reflection by individual student

On-time submission of a decent reflection earned two points, which was included in the total 60 points of the capstone project. One faculty member, RL, evaluated whether the reflection met the requirements specified in the common domain rubric. If the requirement was not met, an appropriate point deduction (< 1 point) might be applied. If a student failed to submit a reflection, no points would be earned.

#### 2.4.3 Performance on capstone diseases in the block 4 examination of the physiology course

To further evaluate the outcome of students’ self-learning on capstone diseases, we monitored performance on exam questions directly tied to the assigned capstone diseases in the Block 4 examination of the Physiology course, as shown in **Table 1**.

### 2.5 Collection of student feedback

To permit student evaluation and collect feedback on the joint capstone project, an anonymous Qualtrics survey was conducted after the final presentations. There were 9 survey items, for which students responded on a Likert scale defined as: strongly disagree = 1, disagree = 2, neutral = 3, agree = 4, or strongly agree = 5. The survey also included one multiple-choice question to estimate the amount of time students spent on the project and one open-ended question soliciting student suggestions for improvement of future joint capstone projects. The survey questions are provided in Information.

### 2.6 Collection of student reflection and qualitative analysis

Each student’s reflection was collected by Microsoft Form. The reflection exercise asked students to consider and respond to one of the following prompts centered on the:.

1. Team.

 a. What were some things your teammates did that helped you to learn or overcome obstacles?

2. Student.

 a. What did you enjoy most about this project? What did you enjoy least?

 b. What would you do differently if you were to complete this project again?

3. Project.

 a. What were some advantages and disadvantages of the combined PHYS & PHARM project?

4. Future Practice.

 a. How do you think this Capstone Project relates to real-world situations and problems?

 b. How will you use what you have learned in this Capstone Project in the future?

Students’ reflections were narratively analyzed, and themes were extracted.

### 2.7 Statistical analysis

The Student's t-test was used for the comparisons of students' performances on the joint capstone projects or the Physiology Block 4 Exam, with p < 0.05 as significantly different. Average ratings on survey items were calculated.

## 3 Results

### 3.1 Student performance

There were 7 teams on each campus. Team performances were assessed based on rubrics. As shown in **Table 4**, as a whole class, the mean score of the physiology domain was 18.9 points, which was 94.5% of a total of 20 points; the mean of the pharmacology domain was 19.8 points, which was 99% of a total of 20 points; the average of the common domain was 19.7 points, meaning 98.6% of a total of 20 points. Average scores were higher than 93.5% in all three domains.

**Table 4:** Performances of joint capstone project (mean ± SD)(%).

Campus	N	Teams	Physiology domain points (% out of 20)	Pharmacology domain points (% out of 20)	Common domain points (% out of 20)
Colorado	40	7	19.2 ± 1.2 (96.0%)	19.6 ± 0.5 (98%)	19.6 ± 0.4 (98.0%)
Utah	32	7	18.7 ± 1.5 (93.5%)	20.0 ± 0 (100%)	19.8 ± 0.3 (99.1%)
Total	72	14	18.9 ± 1.3 (94.5%)	19.8 ± 0.4 (99%)	19.7 ± 0.4 (98.6%)

Note: p> 0.05 between the Colorado and the Utah cohorts.

Furthermore, there were no significant differences between the CO and the UT cohorts in all three domains.

### 3.2 Performances in the block 4 exam of the physiology course

In the Physiology course, because 2 students withdrew, there were 39 students on the Colorado campus and 31 students on the Utah campus who took the Block 4 Exam. These two withdrawn students still attended the Pharmacology course and finished the joint-capstone projects.

Among 14 team-selected capstone diseases in 2022 Spring, only 3 diseases were simply introduced by teachers in the Physiology course in the Fall 2021 or Blocks 1–3 in the Spring 2022, and none were covered in the examining Block 4. Students had to learn all 14 diseases thoroughly by themselves from their own capstone projects and their peers’ capstone presentations. There were 19 questions on the pathophysiology of 14 team-selected capstone diseases, over a total of 30 questions in the Block 4 exam of the Physiology course, accounting for 6.2% of the total 308 points for the Physiology course, as shown in **Table 1**.

Therefore, students’ performance on the 19 questions related to the capstone diseases represented the effectiveness of self-learning, while performance on the 11 questions not related to capstone diseases indicated knowledge gained from teacher-taught content.

As shown in **Table 5**, the mean score of 19 questions related to capstone diseases was not significantly different from the mean performance on the 11 questions not related to capstone diseases (70.31 vs 69.78, p > 0.05). Furthermore, there were no significant differences in the performances between students in the Colorado campus and students in the Utah campus on any questions.

**Table 5:** Comparison between performances on questions related to Capstone diseases and performances on questions not related to Capstone diseases.

Campus	N	Teams	Performance on 19 questions related to capstone projects (Mean ± SD) (Self-learned)	Performance on 11 questions not related to capstone projects (Mean ± SD) (Teacher-taught)
Colorado	39	7	69.21 ± 20.58	69.82 ± 22.62
Utah	31	7	71.68 ± 20.71	69.73 ± 19.64
Total	70	14	70.31 ± 20.36	69.78 ± 19.85

### 3.3 Student feedback

The evaluation survey collected 72 responses (100% response rate). Students evaluated 9 items, as shown in **Table 6**. The average ratings for items 1–8 were 4.0 to 4.5 (maximum 5). The majority of students (77.8% - 88.9%) strongly agreed and agreed that the joint capstone project facilitated self-directed learning of the physiologic aspects of diseases, treatments, clinical trials, drug development, and drug selection (items 1–5 and 7). Students rated enhanced integration of physiology, pharmacology, and diseases (item 7) the highest (an average score of 4.5).

**Table 6:** Student Feedback for Joint Capstone Project (N = 72).

Survey items	Strongly agree [N (%)]	Agree [N (%)]	Neutral [N (%)]	Disagree [N (%)]	Strongly disagree [N (%)]	Mean Rating(out of 5)
1: The joint capstone project facilitated self-directed learning of physiology concepts relevant to a disease	30 (41.7)	37 (51.4)	4 (5.6)	0 (0)	1 (1.4)	4.3
2: The joint capstone project facilitated self-directed learning of standard treatment for a new disease	35 (48.6)	33 (45.8)	4 (5.6)	0 (0)	0 (0)	4.4
3: The joint capstone project facilitated self-directed study of clinical trials to interpret drug efficacy and safety data	31 (43.0)	31 (43.0)	9 (12.5)	1 (1.4)	0 (0)	4.3
4: The joint capstone project facilitated understanding of the drug development process	17 (23.6)	39 (54.2)	13 (18.1)	3 (4.2)	0 (0)	4.0
5: The joint capstone project facilitated learning how to choose a new drug over standard treatment based on a patient’s condition and the characteristics of the drug	20 (27.8)	39 (54.2)	9 (12.5)	3 (4.2)	1 (1.4)	4.0
6: The joint capstone project helped me work and learn as a team member	36 (50.0)	25 (34.7)	8 (11.1)	2 (2.8)	1 (1.4)	4.3
7: The joint capstone project facilitated my understanding of how physiology, pharmacology, and clinical medicine are connected	46 (63.9)	18 (25.0)	7 (9.7)	1 (1.4)	0 (0)	4.5
8: The joint capstone project enhanced my presentation skills	28 (38.9)	31 (43.1)	10 (13.9)	3 (4.2)	0 (0)	4.2
9: Instead of the joint capstone project, I would have preferred a separate physiology project and a separate pharmacology capstone project.	2 (2.8)	2 (2.8)	5 (6.9)	16 (22.2)	47 (65.3)	1.6

Likert scale response options were assigned point values as follows: strongly disagree = 1, disagree = 2, neutral = 3, agree = 4, or strongly agree = 5.

The joint capstone project promoted teamwork with an average rating of 4.3 (item 6).

Finally, this capstone project enhanced students’ presentation skills, with a rating of 4.2 (item 8).

Eighty-seven percent of students did not prefer the separate physiology and pharmacology capstone project (item 9), with an average rating of 1.6 out of 5.

### 3.4 Project completion time

Teams selected their projects at the end of January 2022, and then the teams had 1 month to finish their draft slides. Within one week, faculty members provided written feedback on their draft, indicating which elements the team missed. The team had 3 weeks to finalize their slides. Then each team will gave a 25-minute oral presentation and a 10-minute session to answer questions in person in the classroom in the last 2 weeks of the Spring semester in April 2022.

**Figure 1:**
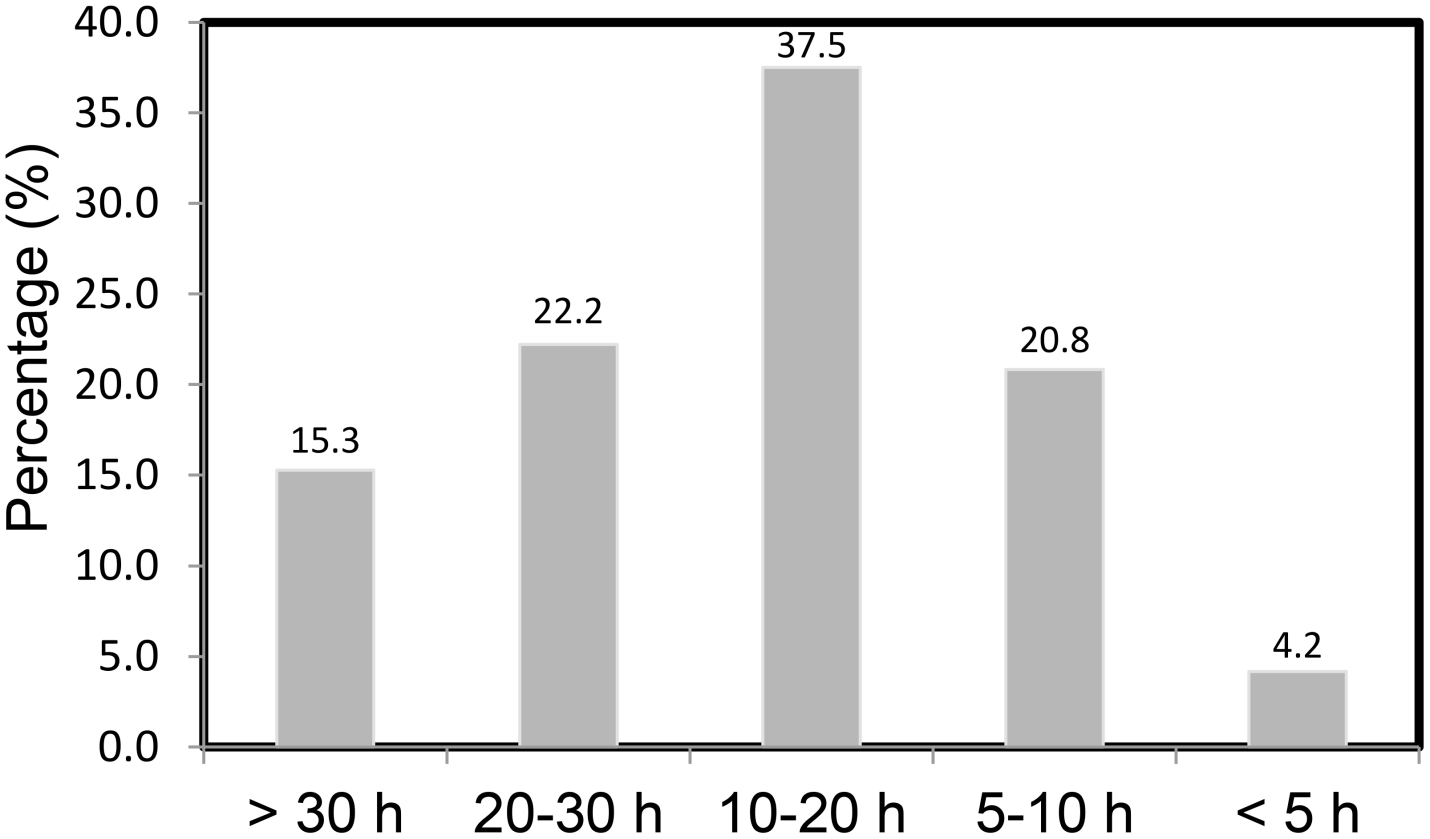
Joint capstone project completion time (N = 72). H, hours.

The survey asked students to estimate how much time they spent on this project. As shown in **Figure 1**, 37.5% of students used 10–20 hours, and the majority of students (80.5%) spent 5–30 hours.

Thirty-seven percent of students spent cumulatively 10–20 hours on the project, while 22.2% and 20.8% of students used 20–30 hours and 5–10 hours, respectively. Some students (15.3%) spent more than 30 hours.

### 3.5 Student recommendations

There were 35 comments (46.7%) in response to the open-ended evaluation survey item soliciting suggestions for improvement of the future joint capstone project. The majority of the comments fell into one of the three categories:

1. Disparate effort invested by individual team members.
2. A team member only knew his/her responsible parts, for example, either physiology or pharmacology, but was unfamiliar with all project sections.
3. Presentation time could be lengthened to ≥ 30 min instead of 25 min to permit presenting in more depth.

### 3.6 Reflections of students

Among individual reflections of 72 students, frequently mentioned themes included integration, teamwork, self-learning, advantages of a joint capstone project over two separate capstone projects, realization of limitations of pharmacology, and future applications. Some quotes from student reflections are summarized in **Table 7**.

**Table 7:** Examples of quotes from students’ reflections.

Themes	Quotes from students’ reflections
Integration	“This project helped to take the learned information, apply it to a disease process, and be able to understand the pathophysiology. The foundations of pharmacology played into physiology because in physiology, we learn how the body normally functions, and how you can manipulate the body’s physiology via pharmacology to treat diseases or produce a desired outcome.” One of the advantages is “Utilizing an interdisciplinary approach to assess the effectiveness of a given treatment.” “What I enjoyed most about this capstone project was the continuity of learning between the physiology and pharmacology principles applied to a specific disease. Diving into the pathophysiology first made understanding drug mechanisms of action and effects more meaningful.” “I felt this project helped me develop the ability to apply basic science concepts across disciplines and the primary literature on the topic. After this capstone, I feel much more confident in my ability to understand a complicated disease process and its treatment, review current literature, and then evaluate how that can be integrated into the current treatment or management approach.” This project “helped evaluate the new drug when in comparison to other established treatments, it challenged me to further understand the disease, as learning the mechanisms and the advantages/disadvantages of other drugs helped me build a better understanding of the disease.”
Teamwork – communication and support	“We were able to teach one another about our sections and were sharing resources to help fill in where someone might have been confused.” “My teammates were very encouraging and helped me with my fear of public speaking. We were able to practice multiple times as a team so that I could be more comfortable talking in front of an audience. They offered advice such as “act like you are talking to one person in the room”. This really helped me and is something I will take with me to future projects.” “My favorite had to have been learning how to interpret clinical trial data and working with all of my group members to pull the final product together.” “Being able to present alternative treatments to a patient with all the pros and cons acts to strengthen trust between the patient and their physician.”
Self-Directed Learning (SDL)	“I needed to do a little extra digging to decide what material I would like to emphasize and teach my classmates during the formal presentation!” “In hindsight (and reflecting), this project helped further my own critical thinking skills. For instance, I was actively using my own understanding of genetic and biochemical implications to understand the pathophysiology of a disease.”
Advantages of joint capstone projects over separate 2 projects	“I appreciate the fact that it is a combined presentation this year. By combining the two presentations together, it gave us an opportunity to recognize how these two classes can be integrated together. With the capstone presentation, we were able to take what we learned in class a step further and figure out how the drug can help alleviate certain diseases, as well as its effects on certain body systems. Another advantage I saw with having two capstone projects combined was that it did give us more time to review content outside of this class and alleviate the stress of having two separate projects to worry about.” “Time-saving in combining two related topics for one project.”
Realization of the limitations of pharmacology	“The major idea I took away from this Capstone was that medicine and pharmacology are very messy and not as straightforward as I thought it was. Specifically, the clinical trials of almost every drug had issues, whether it was the unanticipated outcomes of the drug or the lack of diverse representation in the trials.”
Future application	“As future physicians, we are pledging ourselves to a life of constant learning and understanding of the new treatment options that are coming out daily for a variety of diseases. In this way, the capstone project was an exercise for us that helped to mimic real-world situations that we will experience as physicians.” “Essentially, future conversations with patients are like presenting a new drug therapy to the MSBS class, including fielding difficult questions about the drug. I believe this project has enabled us to quickly find major points about the drug that are pertinent to our patient, and then be able to present that information concisely.” “Another way this project relates to real-world situations and problems is that new drugs are continually developed, and this will continue to shift the rhetoric of treatment towards personalized medicine, where people with a similar disease may have different treatment options given the possible adverse drug reactions someone can experience or even the efficacy of the medication.” “As a provider, I will have to collaborate with other providers and staff in day-to-day operations, such as scheduling and utilization of resources. I will need to collaborate and/or consult with other providers, patient families, and patients themselves about patient care, particularly in complex cases.”

## 4 Discussion

This project helped students apply what they learned in the class to a non-teacher-taught disease and to select appropriate treatment, comparing a non-teacher-taught new drug to the standard care. Students did great as a team for the joint capstone projects and performed excellently individually on the self-learned pathophysiology of capstone diseases in the Block 4 exam of the Physiology course. Student performance, feedback, and self-reflections demonstrated successful enhancement of physiology (pathophysiology), pharmacology, and disease integration within a joint Physiology-Pharmacology capstone project. This capstone project fostered self-directed learning and teamwork as well.

Integration is an important part of medical education. Integration includes horizontal integration between parallel disciplines, such as physiology and pharmacology, and vertical integration between basic medicine and clinical medicine (Youm et al., 2024). When students learn different disciplines, such as physiology, pharmacology, etc., and different organ systems, such as the cardiovascular system, pulmonary system, etc., they have to make connections among disciplines and systems because the human body functions as a whole. Integration can occur at different levels, and many experts recommend early integration between basic and clinical science (Kulasegaram et al., 2013; Roberts et al., 2023). According to Harden’s 11-step integration ladder, our joint capstone projects could belong to Step 11: Transdisciplinary integration (Harden, 2000; Hamsaveena et al., 2022). For Step 11, “This is the highest level of integration. The emphasis is here on knowledge as exemplified in real world. It is the responsibility of the student to integrate all that is learnt in different disciplines when a real-life situation is encountered” (Hamsaveena et al., 2022). Our joint Physiology-Pharmacology capstone project is disease-centered learning, mimicking patient-centered learning (Anderson et al., 2020). The ability to integrate self-learned capstone diseases into physiology was shown in our students’ good performances in the summative Block 4 examination of the Physiology course. Medical residents rate anatomy, physiology, and pharmacology as the top basic science subjects preparing them for clinical rotations and residency (Chan et al., 2012). Therefore, this joint capstone project emphasizes the linkage among physiology, pharmacology, and diseases at an early step on the path to becoming a physician. The MSBS students were still in the premed stage and had not entered medical school yet. However, the joint capstone projects opened students’ eyes, taught them the limitations of pharmacology, and helped them understand the non-teacher-taught diseases in depth. In the self-reflection, students stated that after the joint capstone project, they were much more confident in their ability to understand a complicated disease process and its treatment, review current literature, and then evaluate how that can be integrated into the current treatment or management approach.

Self-directed learning (SDL) is a skill needed for premedical students, medical students, and physicians, as alluded to in the premed competencies (Commitment to Learning and Growth) and the core competencies for medical residents (Practice-Based Learning and Improvement) (The Premed Competencies for Entering Medical Students; ACGME, [[NoYear]]). SDL is the opposite of teacher-directed learning and is recommended in the modern medical curriculum. Knowles identified the competencies of SDL in 1975 (Knowles, 1975). Self-directed learning permits students’ freedom with autonomy, flexibility of learning, and responsibility for mastering new knowledge and skills (Charokar and Dulloo, 2022). “Self-directed learning eventually empowers the medical students to develop the competencies for lifelong learning, which is one of the five roles expected from a Competent Indian medical graduate” (Charokar and Dulloo, 2022). In our capstone projects, students demonstrated their effectiveness in self-learning by their equivalent performances on questions related to capstone diseases (SDL) compared to their performance on questions not related to capstone diseases (Teacher-taught) in the Block 4 examination of the Physiology course.

To become a self-directed learner, self-reflection, one of the many competencies, should be acquired (Knowles, 1975; Charokar and Dulloo, 2022). Considering the continuous development of science, technology, and medicine, and the occurrence of new diseases, it is paramount for healthcare providers to continuously learn new information throughout their careers. Effective self-directed learning requires training, just like other skills in medical school (Ginzburg et al., 2021). In this joint capstone project, students need to not only know “what this is” but also understand “why this is” by self-learning the pathophysiology of non-teacher-taught diseases and treatments, synthesizing the available literature, generating presentation slides, presenting and teaching MSBS students, and answering faculty and peer questions during the presentation. Effective self-directed learning seeks this kind of critical reasoning (Ginzburg et al., 2021). As in student reflections, one student said, “As future physicians, we are pledging ourselves to a life of constant learning and understanding of the new treatment options that are coming out daily for a variety of diseases. In this way, the capstone project was an exercise for us that helped to mimic real-world situations that we will experience as physicians”.

Teamwork is essential in healthcare. This has been indicated in the premed competencies (Teamwork and Collaboration) and the core competencies for medical residents’ skills (Interpersonal and Communication Skills) (The Premed Competencies for Entering Medical Students; ACGME, [[NoYear]]). Effective teamwork improves patients’ outcomes (Babiker et al., 2014). By doing the joint capstone project, student teams divided duties, communicated with each other, and combined each member’s knowledge. This process requires communication, understanding, mutual help, and cooperation. Working as a longitudinal team (the same team) throughout the 10-month program developed highly effective teams and precious friendships within the team. Some student pointed out that the teams helped them overcome the fear of public speaking and enhanced their communication skill in their reflection. Students foresee that in the future, “As a provider, I will have to collaborate with other providers and staff in day-to-day operations” and “Be able to present alternative treatments to a patient with all the pros and cons”.

To our knowledge, this is the first joint Physiology-Pharmacology capstone project implemented in a premedical master’s program. Capstone project exposes real-world diseases to premed students, fostering self-learning and the skill of synthesizing information, which students may not learn from the didactic lectures.

There are some limitations to this project. One limitation was that the time spent on the previous year’s two separate capstone projects (Physiology capstone project and Pharmacology) was not investigated. Although time-saving could not be directly compared between the year 2021 and the year 2022, due to reduced workload, the joint capstone project should have saved more time compared to the previous year’s two separate capstone projects. This makes sense because the pathophysiology and common domain overlapped two separate capstone projects, and the thesis written previously in the pharmacology capstone project was omitted from the joint capstone project. Another limitation was that it is difficult to compare the outcome of self-learning and integration between the year 2021, with two separate physiology and pharmacology capstone projects, and the year 2022, with a single joint capstone project, because student cohorts, capstone diseases, and new drugs were different. In addition, many students felt that 25 minutes for a presentation was not enough. For a more in-depth presentation, time could be increased to 30–35 minutes in the future. Further, some students thought they were only familiar with the sections they were responsible for and knew less about the parts they were not assigned, despite the data supporting widespread agreement with improved integration. In the future, one student can be in charge of a small part of physiology and a small part of pharmacology simultaneously. Students commented that some of their team members did not contribute as much as others. In the future, peer evaluation within one team may be necessary to enhance individual contributions. In addition, due to the limited number of physiology and pharmacology faculty, only one faculty member assessed each domain. In the future, at least two faculty members for each domain should be assigned to facilitate fair and objective grading. Finally, the long-term effects of this capstone project on students in their future studies in medical schools are beyond the current project, but it is a good direction for our future study.

In conclusion, a joint Physiology-Pharmacology capstone project in a premedical master’s program may effectively facilitate the integration of basic science and clinical medicine, teamwork, and self-directed learning. In the future, to enhance and motivate each individual’s contribution in this project, peer evaluation can be implemented. In addition, following up with students on the long-term effects of the joint capstone project is warranted.

## Data Availability

The raw data supporting the conclusions of this article will be made available by the authors, without undue reservation.
