# Supplementary material for: Introduction of a joint physiology-pharmacology capstone project in a premedical master’s program
Source: Front Physiol. 2026 Jul 1;17:1871233. doi: 10.3389/fphys.2026.1871233 (PMC13368504; doi:10.3389/fphys.2026.1871233)

**Supplemental information**

**Table S1. Disease-Drug Pairs**

|  | **Diseases** | **New drugs** | **FDA approval** |
| --- | --- | --- | --- |
| 1 | Alzheimer’s disease | aducanumab-avwa | 6/7/2021 |
| 2 | asthma | benralizumab | 11/14/2017 |
| 3 | carcinoid syndrome | telotristat ethyl | 2/28/2017 |
| 4 | chronic heart failure | vericiguat | 1/19/2021 |
| 5 | chronic hepatitis C virus | glecaprevir and pibrentasvir | 8/3/2017 |
| 6 | chronic obstructive pulmonary disease (COPD) | revefenacin | 11/9/2018 |
| 7 | COVID-19 | sotrovimab | EUA 5/26/2021 |
|  |  | nirmatrelvir with ritonavir | EUA 12/22/2021 |
|  |  | baricitinib | 11/19/2020 |
|  |  | tocilizumab | 6/24/21 |
| 8 | Cushing’s disease | osilodrostat | 3/6/2020 |
| 9 | cystic fibrosis | elexacaftor/ivacaftor/tezacaftor | 10/21/2019 |
| 10 | Duchenne muscular dystrophy | casimersen | 2/25/2021 |
| 11 | endometriosis | elagolix sodium | 7/23/2018 |
| 12 | glaucoma | netarsudil | 12/18/2017 |
| 13 | Huntington’s disease | deutetrabenazine | 4/3/2017 |
| 14 | Lambert-Eaton myasthenic syndrome (LEMS) | amifampridine | 11/28/2018 |
| 15 | septic or other distributive shock | angiotensin II | 12/21/2017 |
| 16 | short stature due to inadequate secretion of endogenous growth hormone | lonapegsomatropin-tcgd | 8/25/2021 |
| 17 | type 2 diabetes mellitus | ertugliflozin | 12/19/2017 |

Abbreviations: FDA = [U.S. Food and Drug Administration; EUA = Emergency Use Authorization](https://www.fda.gov/)

**Supplemental information**: evaluation survey questions.

**Survey questions**

Q1. The joint capstone project facilitated self-directed learning of physiology concepts relevant to a disease.

1. Strongly agree
2. Agree
3. Neutral
4. Disagree
5. Strongly disagree

Q2. The joint capstone project facilitated self-directed learning of standard treatment for a new disease.

1. Strongly agree
2. Agree
3. Neutral
4. Disagree
5. Strongly disagree

Q3. The joint capstone project facilitated self-directed study of clinical trials to interpret drug efficacy and safety data.

1. Strongly agree
2. Agree
3. Neutral
4. Disagree
5. Strongly disagree

Q4. The joint capstone project facilitated understanding of the drug development process.

1. Strongly agree
2. Agree
3. Neutral
4. Disagree
5. Strongly disagree

Q5. The joint capstone project facilitated learning how to choose a new drug over standard treatment based on a patient’s condition and the characteristics of the drug.

1. Strongly agree
2. Agree
3. Neutral
4. Disagree
5. Strongly disagree

Q6. The joint capstone project helped me work and learn as a team member.

1. Strongly agree
2. Agree
3. Neutral
4. Disagree
5. Strongly disagree

Q7. The joint capstone project facilitated my understanding of how physiology, pharmacology, and clinical medicine are connected.

1. Strongly agree
2. Agree
3. Neutral
4. Disagree
5. Strongly disagree

Q8. The joint capstone project enhanced my presentation skills.

1. Strongly agree
2. Agree
3. Neutral
4. Disagree
5. Strongly disagree

Q9. Instead of the joint capstone project, I would have preferred a separate physiology project and a separate pharmacology capstone project?

1. Strongly agree
2. Agree
3. Neutral
4. Disagree
5. Strongly disagree

Q10. Approximately how much time did you spend on the joint capstone project?

1. More than 30 hours
2. 20-30 hours
3. 10-20 hours
4. 5-10 hours
5. Less than 5 hours

Q11. What suggestions do you have for improving future joint capstone projects?

Supplemental Information: Full Rubrics

**Table S2. PHYS Domain Rubric (20 Points total)**

|  | **Poor/Needs Improvement**  (0 to 70%) | **Approaches Expectations**  (70 to 80%) | **Meets Expectations**  (80-100%) | **Score** | |
| --- | --- | --- | --- | --- | --- |
|  |  |  |  | **%** | **Pts** |
| **Physiology Concepts**  (10 points max) | □ Physiology information is not thorough or concise  □ Includes few pertinent concepts with unclear or incorrect explanations  □ Includes one or fewer physiology concepts and/or all concepts from only one block | □ Physiology information somewhat thorough and concise  □ Includes some pertinent concepts and explains them somewhat clearly and correctly  □ Includes two distinct physiology concepts from at least two different blocks or three or more from only two blocks | □ Physiology information thorough and concise  □ Includes pertinent concepts and explains them clearly and correctly  □ Includes at least three distinct physiology concepts from at least three different blocks |  |  |
| **Pathophysiology Integration**  (5 points max) | □ Explanation of case findings and/or pathophysiology is incorrect and/or does not clearly and/or correctly connects the physiology concepts | □ Explanation of case findings and/or pathophysiology is somewhat correct and/or partially connects the physiology concepts | □ Explanation of case findings and/or pathophysiology is correct and robustly connects the physiology concepts |  |  |
| Case  (5 points max) | □ Case is not very plausible  □ Includes inadequate pertinent symptoms, vitals, physical exam, labs/studies, and/or treatments | □ Case is somewhat plausible  □ Includes some pertinent symptoms, vitals, physical exam, labs/studies, and/or treatment | □ Case is plausible  □ Includes pertinent symptoms, vitals, physical exam, labs/studies, and treatment |  |  |
| **TOTAL(out of 20)** | | | |  |  |

**Table S3. PHARM Domain Rubric (20 Points total)**

|  | **Poor/Needs Improvement**  (0 to 70%) | **Approaches Expectations**  (70 to 80%) | **Meets Expectations**  (80-100%) | **Score** | |
| --- | --- | --- | --- | --- | --- |
|  |  |  |  | **%** | **Pts** |
| **Pharmacology Concepts**  (10 points max) | □ Drug mechanism of action is not thorough or concise  □ Includes few pharmacokinetic parameters with unclear or incorrect explanations  □Adverse drug reactions are not adequately discussed | □ Drug mechanism of action somewhat thorough and concise  □ Includes some pharmacokinetic parameters and explains them somewhat clearly and correctly  □ Adverse drug reactions are discussed somewhat adequately | □ Drug mechanism of action thorough and concise  □ Includes pharmacokinetic parameters and explains them clearly and correctly  □Adverse drug reactions are thoroughly and adequately discussed |  |  |
| **Review of Drug Development Literature**  (3 points max) | □ Background relevant to history of drug development is not discussed  □ Clinical trial (at least one Phase II or one Phase III) is not adequately discussed | □ Background relevant to history of drug development is somewhat adequately discussed  □ Clinical trial (at least one Phase II or one Phase III) is somewhat adequately discussed | □ Background relevant to history of drug development is adequately discussed  □ Clinical trial (at least one Phase II or one Phase III) is adequately discussed |  |  |
| **Standard of Care**  (7 points max) | □ Current standard of care is not discussed  □ Chosen drug’s value in disease management compared to current standard of care is not discussed | □ Current standard of care is somewhat adequately discussed  □ Chosen drug’s value in disease management compared to current standard of care is somewhat adequately discussed | □ Current standard of care is adequately discussed  □ Chosen drug’s value in disease management compared to current standard of care is adequately discussed |  |  |
| **TOTAL TEAM POINTS (out of 20)** | | | |  |  |

**Table S4. Common Domain Rubric**

**(****20 Points total distributed as total % applied to 10 Points PHYS and 10 Points PHARM)**

|  | **Poor/Needs Improvement**  (0 to 70%) | **Approaches Expectations**  (70 to 80%) | **Meets Expectations**  (80-100%) | **Score** | |
| --- | --- | --- | --- | --- | --- |
|  |  |  |  | **%** | **Pts** |
| **Written Communication and Visual Aids**  (5 points max) | □ Visual aids have significantly too little/much information, are of poor quality, and fail to effectively convey content | □ Visual aids contain too little/much information, are of mediocre quality, and somewhat convey content | □ Visual aids contain appropriate amount of information, are of good quality, and effective at conveying content |  |  |
| **Oral Communication**  (5 points max) | □ The speakers are minimally confident, articulate, and professional  □ Most information read from notes/slides  □ Speakers are unable to adequately respond to questions from faculty & peers | □ The speakers are somewhat confident, articulate, and professional  □ Some of information is memorized but often is read from notes/slides  □ Speakers respond somewhat adequately to questions from faculty & peers | □ The speakers are confident, articulate, and professional  □ Majority of information is memorized (not read from notes/slides)  □ Speakers respond adequately to questions from faculty & peers |  |  |
| **Professional Behavior**  (2 points max) | □ Speakers are casually or inappropriately dressed  □ Disrespectful interactions with team, audience, and/or faculty  □ Two or more assignments were not completed by the due date | □ Speakers are somewhat professionally dressed  □ Somewhat respectful interactions with team, audience, and/or faculty  □ One or more assignments were not completed by the due date | □ Speakers are professionally dressed  □ Respectful interactions with team, audience, and/or faculty  □ All assignments were completed by the due date |  |  |
| **Learning Outcomes**  (2 points max) | □ Presentation does not include 3-5 learning outcomes | □ Presentation includes 3-5 learning outcomes that lack specificity or pertinence | □ Presentation includes 3-5 specific, pertinent learning outcomes |  |  |
| Racial and Ethnic Health Disparities  (2 points max) | □ Racial and ethnic disparities relevant to disease and/or drug treatment are not discussed | □ Racial and ethnic disparities relevant to disease and/or drug treatment are somewhat adequately discussed | □ Racial and ethnic disparities relevant to disease and/or drug treatment are adequately discussed |  |  |
| **Sources and Citations**  (2 points max) | □ Hardly any non-original information is from a credible source and is appropriately cited using APA in text or endnote format  □ Few images are appropriately cited | □ Nearly all non-original information is from a credible source and is appropriately cited using APA in text or endnote format  □ Nearly all images are appropriately cited | □ All non-original content is from a credible source and is appropriately cited using APA in text or endnote format  □ All images are appropriately cited (minimum of URL) |  |  |
| **Timing** (penalty points only) | □ Presentation is less than 22 min and/or over 25 min (-2 pts) | □ Presentation is over 19 min and/or under 28 min (-1 pt), but not within official time | □ Presentation is over 22 min and less than 25 min |  |  |
| **Final Reflection***  (2 points max) | □ Little evidence of connection to real-life  □ Minimal content; lacks analysis, synthesis, and evaluation | □ Connects ideas and thoughts to real-life  □ Substantial content; evidence of analysis, synthesis, and evaluation | □ Evidence of personal reflection that makes connections between learning and collaborative practice  □ Rich in content; insightful analysis, synthesis, and evaluation |  |  |
| **TOTAL POINTS (out of 18 team + 2 individual)** | | | |  |  |
| **PHYS POINTS (out of 9 + 1)** | | | |  |  |
| **PHARM POINTS (out of 9 + 1)** | | | |  |  |

 * Final Reflection is an individual assignment and will be applied to each students’ grade as a separate assignment worth 1 point in the PHYS II Course and 1 point in the PHARM Course (2 points total).

Exemption Approval: IRB #2022-141 from RVU IRB Committee


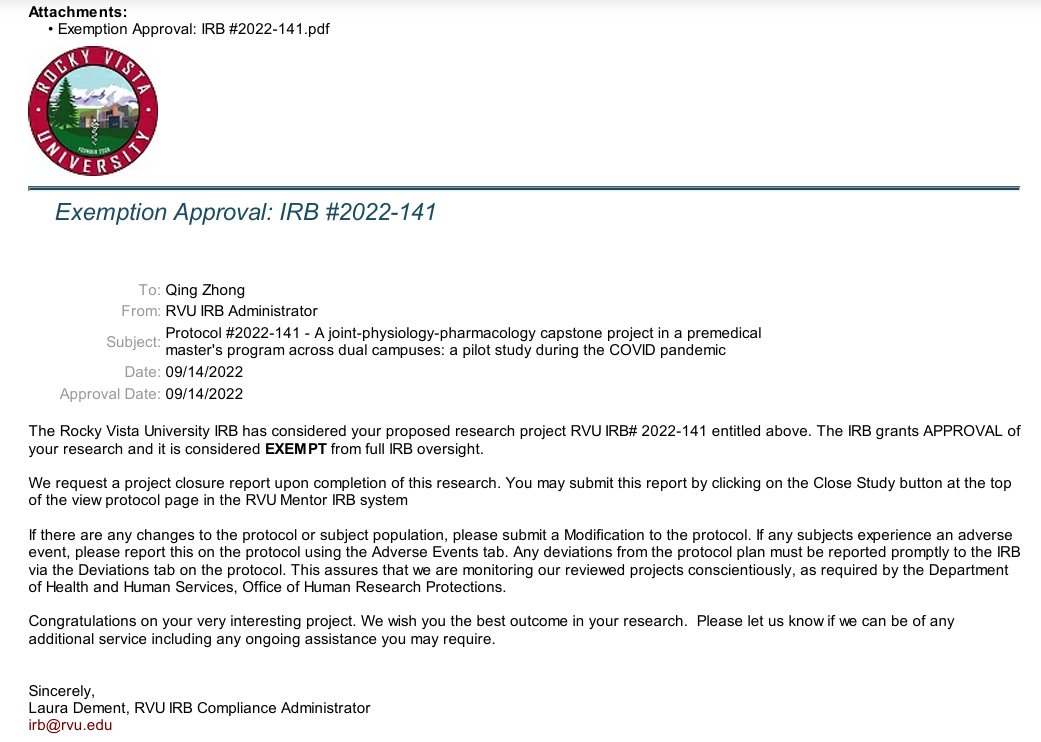

Supplement: Supplementary file 1 [file SupplementaryFile1.docx]
